# Rapid spread of the SARS-CoV-2 Delta variant in some French regions, June 2021

**DOI:** 10.2807/1560-7917.ES.2021.26.28.2100573

**Published:** 2021-07-15

**Authors:** Samuel Alizon, Stéphanie Haim-Boukobza, Vincent Foulongne, Laura Verdurme, Sabine Trombert-Paolantoni, Emmanuel Lecorche, Bénédicte Roquebert, Mircea T. Sofonea

**Affiliations:** 1 MIVEGEC, CNRS, IRD, Université de Montpellier, Montpellier, France; 2 Laboratoire Cerba, Saint Ouen L’Aumône, France; 3 Laboratoire de Virologie, CHU de Montpellier, France

**Keywords:** COVID-19, virus, variant, RT-PCR, epidemiology, statistical modelling, simulations

## Abstract

We analysed 9,030 variant-specific RT-PCR tests performed on SARS-CoV-2-positive samples collected in France between 31 May and 21 June 2021. This analysis revealed rapid growth of the Delta variant in three of the 13 metropolitan French regions and estimated a +79% (95% confidence interval: 52–110%) transmission advantage compared with the Alpha variant. The next weeks will prove decisive and the magnitude of the estimated transmission advantages of the Delta variant could represent a major challenge for public health authorities.

The evolution of severe acute respiratory syndrome coronavirus 2 (SARS-CoV-2) is characterised by the emergence of several variants which have caused major epidemics in the United Kingdom (UK) [1,2], Brazil [3], and South Africa [4]. In May 2021, the Delta variant (Phylogenetic Assignment of Named Global Outbreak (Pango) lineage designation B.1.617.2), first detected in India was designated a variant of concern by the World Health Organization [5] and was associated with an epidemic rebound in the UK [6]. Analyses showed a transmission advantage of ca 66% (95% confidence interval (CI): 28–113%) over the Alpha variant (B.1.1.7) in England [7] and the Delta variant appeared slightly more prone to immune evasion [8]. 

As evident with the emergence of the Alpha variant earlier in 2021, there appears to be a 2-month shift between the French and epidemics in the UK [9,10]. Therefore, it is timely to investigate the potential early spread of the Delta variant in France to devise appropriate public health responses.

## Detection of the SARS-CoV-2 Delta variant

We analysed 9,030 RT-PCR variant-specific screening tests (VirSNiP assay, TIB Molbiol, Berlin, Germany) performed on samples collected in France by the CERBA network of medical analysis laboratories in patients from 5 to 80 years old between 31 May and 21 June 2021 that were positive for SARS-CoV-2. The assay screens for the presence of the E484K, the E484Q, and the L425R mutations (Supplement). A 484K+ /484Q−/452R− profile is consistent with the Beta, Gamma, or Eta variants (B.1.351, P.1, and B.1.525 Pango lineages, respectively), whereas a 484K−/484Q−/452R+ profile is consistent with mainly the Delta variant [11]. While other variants of interest (VOI) or under monitoring (VUM) are consistent with the latter profile – e.g. Kappa VOI or 21C VUM (B.1.427 and B.1.429 Pango lineages, respectively) – sequencing surveys have shown their relative frequency to be negligible in France [12]. To validate the VirSNiP assay, samples already characterized by NGS sequencing (COVIDSEQ Illumina) were tested. Of those tested, 333 samples were found with a 484K−/484Q−/452R+ profile, consistent with the Delta variant, in 329 cases (98.8%). Three cases showed a 452R+ profile with 484K or 484Q mutations uninterpretable (0.9%) and one case showed a 484K−/484Q+/452R+ profile (0.3%). Therefore, the specificity of the VirSNiP assay to define 484K−/484Q−/452R+ profile as the Delta variant is 98.8%. Of 342 tests with a 484K−/484Q−/452R+ VirSNiP profile, the sequencing assay showed 329 Delta variants, two variants B.1.1.7/452R, two variants 20D and 10 sequences that were uninterpretable because of NGS coverage less than 98.5%. Calculated sensitivity was 96.2% when including uninterpretable sequences and 99% when discarding the 10 uninterpretable sequences. These tests were performed on samples from different French regions, with the majority from the Île-de-France (the Paris area; 50%; 4,483/9,030 tests; Supplementary Figure S1) and were mostly from non-hospitalised individuals (93%; 8,404/9,030 tests; Supplementary Table S1).

## Variant-screening test results

Using a multinomial regression model [13], we found that samples bearing the L452R mutation but not the E484K or the E484Q mutation (consistent with the Delta variant), tended to increase in the Hauts-de-France, Île-de-France, Normandie, and Provence-Alpes-Côte d'Azur regions compared with our reference, which were samples lacking the three mutations, consistent with the Alpha variant (Table).

**Table:** Factors associated with the detection of potential SARS-CoV-2 variants compared with the Alpha variant, as assessed by relative risk ratios using a multinomial log-linear model, France, 31 May–21 June 2021 (n = 8,190)

Factor		RRR per variant					
		Beta/Gamma/Eta variants^a^		Delta variant^a^		Other variants^a^	
		RRR	95% CI	RRR	95% CI	RRR	95% CI
Age		1.07	1.00–1.10	NS	0.94–1.20	NS	0.98–1.10
Sample origin	Hospital samples	Ref.					
	Non-hospital samples	0.62	0.49–0.80	NS	0.64–1.60	NS	0.71–1.10
Interaction between the sampling region and the calendar date of sampling	Normandie	NS	0.97–1.40	1.72	1.30–2.20	1.40	1.20–1.60
	Hauts-de-France	1.29	1.00–1.60	2.00	1.50–2.70	1.52	1.30–1.80
	Ile-de-France	1.19	1.10–1.30	2.23	1.90–2.60	1.50	1.40–1.60
	Provence−Alpes−Côte d'Azur	NS	0.75–1.30	1.92	1.40–2.70	NS	0.92–1.30
	Centre-Val-de-Loire	NS	0.56–1.20	NS	0.51–1.70	1.29	1.00–1.70
	Other regions	NS	0.71–1.00	NS	0.70–1.30	1.40	1.20–1.60

CI: confidence interval; NS: non-significant; RRR: relative risk ratio; SARS-CoV-2: severe acute respiratory syndrome coronavirus 2; VOI: variant of interest; VUM: variant under monitoring.

^a^ A 484K+ /484Q−/452R− profile is consistent with the Beta, Gamma, or Eta variants (B.1.351, P.1, and B.1.525 Pango lineages, respectively), whereas a 484K−/484Q−/452R+ profile is consistent with the Delta variant plus other variants of negligible incidence such as Kappa VOI or 21C VUM (B.1.427 and B.1.429 Pango lineages, respectively). Other variants include: 20I/501Y.V1, 20I/484K, 20I/484Q, 20I/452R, 20B/681H (B.1.1.318), 20A/440K (B.1.619), 20A/477N (B.1.620), 20C/484K, 20C, 20D, 20D/452R, 20D/484K, which were sequenced by the CERBA laboratory.

In the model, screening tests consistent with an Alpha variant are the reference for the RRR. See [13] for details about the methods used. Our analyses were performed on 8,190 samples since 840 samples with missing values for the factors considered in the model were not included. The level of significance is p < 0.05.

Samples collected outside hospital settings were less likely to originate from Beta/Gamma/Eta variants than from the Alpha variant. Finally, in the Hauts-de-France and Île-de-France regions, we found a temporal increase of Beta/Gamma/Eta variants compared with the Alpha variant. In most regions, we found a temporal increase of ‘other’ test results compared with the Alpha variant. This trend is more difficult to interpret since these tests may correspond to any variant.

## Transmission advantages

**Figure 1:**
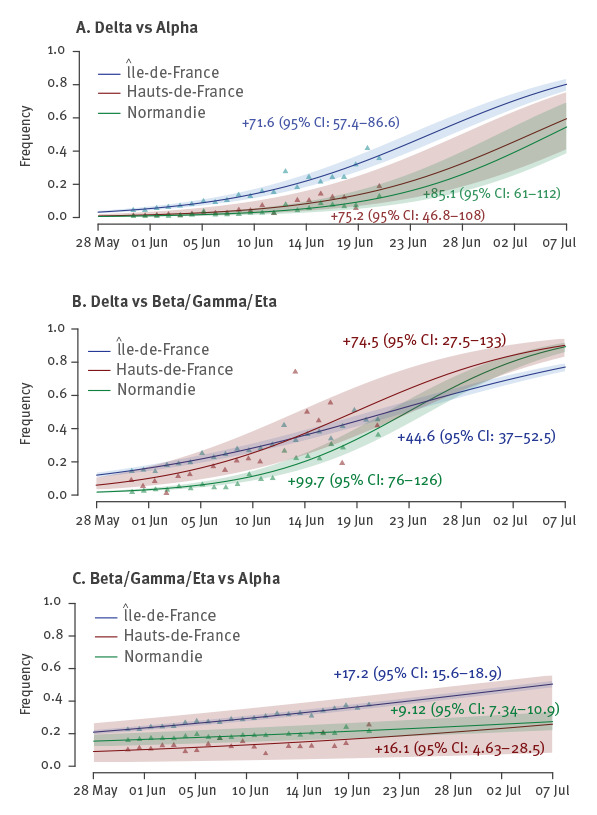
Change in relative frequency and estimated transmission advantage between SARS-CoV-2 Alpha, Beta/Gamma/Eta, and Delta variants in Hauts-de-France, Île-de-France, and Normandie, France, 31 May–21 June 2021

CI: confidence interval; SARS-CoV-2: severe acute respiratory syndrome coronavirus 2.

To further quantify the temporal trends, we performed a statistical analysis using a logistic growth model following previous studies [1,2,9,14] and pairwise comparisons between the three main interpretations of the test results, i.e. consistent with infections by Delta, Beta/Gamma/Eta, or Alpha variants. Between 31 May and 21 June, the Delta variant was found to have a mean transmission advantage over the Alpha variant of greater than 79% (95% CI: 52–110%) in Hauts-de-France, Île-de-France, and Normandie (Figure 1A). In these three regions, we also found a significant transmission advantage of the Delta variant over the Beta/Gamma/Eta variants (Figure 1B). Finally, consistently with our previous results [13], we find that Beta/Gamma/Eta variants have a significant, but smaller, transmission advantage over the Alpha variant in these regions (Figure 1C). Transmission advantages tend to decrease with time (Supplementary Figure S3), which could be linked to a delay in collecting the cases or a change in selection pressures.

The triangles indicate the generalised linear model-fitted values and the line indicates the output of the logistic growth model estimation. The shaded area represents the 95% CI. The estimated transmission advantages are shown in the panels.

## Doubling times

Using the incidence of Delta variant-positive tests (484K−/484Q−/452R+; Supplementary Figure S4), we computed epidemic doubling times. When using a 14-day interval, we find low values such as 9.8 days (95 CI%: 6.3–22.6) in Île-de-France and 10.5 days (95 CI%: 6.9–21.9) in Hauts-de-France. However, in Île-de-France, this doubling time strongly increases over time and a 21-day interval yields a median doubling time of 26.9 days (Supplementary Figure S5). This pattern could be explained by a delay in case reporting or, conversely, by an initial over-representation of superspreading events.

## Modelling epidemic scenarios

**Figure 2:**
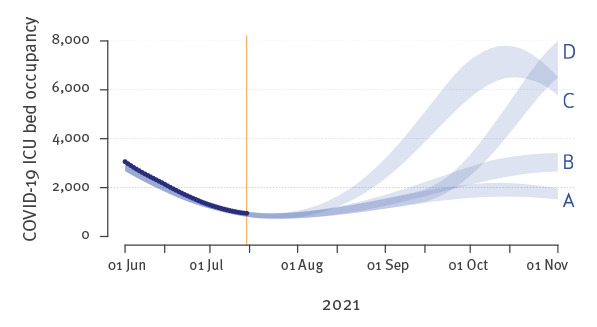
COVIDSIM^a^ projections of the COVID-19 epidemic for different vaccine rollout and back-to-school effect scenarios, France, June 2021

COVID-19: coronavirus disease; ICU: intensive care unit.

^a^ COVIDSIM model projections were performed according to [15].

Using an epidemiological model of the French epidemic [15], we investigated the potential consequences of our results in the medium term (4 months ahead). In Figure 2, we explored four scenarios (A, B, C, and D) that differ by arbitrary levels of vaccine rollout and background contact rate. In scenarios A and B, we assume no transmission increase (other than the transmission advantage of the Delta variant) whereas in scenario C and D, we imposed respectively a 10% from 14 July 2021 and a 20% from 1 September 2021 increase in background contact rate (compared with June 2021), in order to explore the impact of major summertime loosening of preventive measures and back-to-school effect. In scenarios A and C, the vaccine rollout is assumed to result in a 66% coverage of the whole population on 1 September, whereas in scenario B, we consider a slower vaccination pace, resulting in ca 61% vaccination coverage by the end of the summer (see details in the Supplementary Methods). We find that the Delta variant has the potential to initiate an epidemic rebound by the end of the summer that could be amplified by a slowdown in vaccine rollout (Figure 2). Even more, a major increase in background infectious contact rate in September could lead to an intensive care unit (ICU) overload (scenarios C and D). In the absence of any additional mitigation, the residual coronavirus disease (COVID-19) hospital mortality for the second half of 2021 in scenarios A and B remains limited and lies between 10 and 15% of the current COVID-19 death toll in France. The trend of scenario C or D would, however, call for vigilance and mitigation by the end of the summer if ICU overload is to be avoided and hospital mortality capped.

Scenario A: Fast vaccine rollout (66% of the whole French population is assumed fully vaccinated on 1 September) and constant background contact rate (the transmission increase is solely due to the spread of the Delta variant, assumed to be 70% more transmissible than the Alpha variant).

Scenario B: Slower vaccine rollout (−5% coverage by the end of the summer compared with scenario A) and constant background contact rate.

Scenario C: Fast vaccine rollout and strong summertime loosening of preventive measures (+10% increase in background contact rate from 14 July compared with June 2021).

Scenario D: Fast vaccine rollout and strong back-to-school effect (+20% increase in background contact rate from 1 September compared with June 2021).

The blue stripes represent the current nationwide COVID-19 ICU bed occupancy according to the three scenarios. The width of each stripe corresponds to the range spanned by 95% of the associated simulations. The turquoise dots are the rolling 7-day average of national COVID-19 ICU bed occupancy. The orange vertical line indicates the day the simulation was performed.

## Ethical statement

This study has been approved by the Institutional Review Board of the CHU of Montpellier and is registered at ClinicalTrials.gov with identifier NCT04738331.

## Discussion

Screening for SARS-CoV-2 mutations carried by the Delta variant on samples collected between 31 May and 21 June 2021 indicates that this variant might be spreading rapidly in French regions. According to our estimates, the Delta variant may have already been more frequent than the Alpha variant in Île-de-France by the end of June 2021.

There are several potential biases to this analysis, which we attempted to correct. First, since our dataset continually gathers results from local laboratories, there can be delays in data centralisation. To address this, we only analysed the data up to 21 June 2021 and ignored the data collected between 22 and 28 June. Another potential issue relates to the specificity of the test used. Our estimated frequency of the Delta variant was ca 4% of the positive tests on 8 June. This is consistent with the outcome of national sequencing studies (0.2% on 11 May 2021 [16]) and the doubling times comparable to those we estimated in the early stages of the Delta variant epidemic in Île-de-France (Supplementary Figure S5). Regarding the sampling scheme, the French authorities have announced strict monitoring of the spread of the Delta variant, which could artificially enrich the data with Delta variant-positive tests. We do not know the context in which most tests were performed, except for in a hospital setting and mainly for hospitalised patients. We expect hospital samples to be less impacted by a potential bias related to contact tracing because, aside from nosocomial infections, the contacts of a hospitalised person are unlikely to be hospitalised. Indeed, we find no association between the hospitalisation status and the Delta variant in the multinomial model.

The estimated transmission advantages of 79% (95% CI: 52–110%) the Delta variant over the Alpha variant are in line with that estimated in the UK [7] and from the analysis of the number of variant sequences in the GISAID database [17]. Consistent with our earlier findings from April 2021 [13], we also find that Beta/Gamma/Eta variants have a transmission advantage over the Alpha variant.

These results have public health implications because they imply that variant Delta could shift epidemic trends. In absence of specific interventions and based on the current vaccine rollout, our model tailored to the French epidemic context suggests that this could cause another COVID-19 wave starting in August 2021. The magnitude and exact timing of this wave would depend on the exact transmission advantage of the variant, the increase in vaccination coverage and a potential loosening of physical distancing and indoor mask-wearing as well as back-to-school effect. This picture could be further complicated by the simultaneous spread of variants Beta, Gamma, and Delta.

## Supplementary Data

786000 Supplement
