# Supplementary material for: Rapid spread of the SARS-CoV-2 Delta variant in some French regions, June 2021
Source: Euro Surveill. 2021 Jul 15;26(28):2100573. doi: 10.2807/1560-7917.ES.2021.26.28.2100573 (PMC8284044; doi:10.2807/1560-7917.ES.2021.26.28.2100573)
Supplement: Supplement [file 21-00573_ALIZON_Supplement.pdf]

This supplementary material is hosted by Eurosurveillance as supporting information alongside the article Rapid spread of the SARS-CoV-2 Delta variant in some French regions, June 2021 on behalf of the authors who remain responsible for the accuracy and appropriateness of the content. The same standards for ethics, copyright, attributions and permissions as for the article apply. Supplements are not edited by Eurosurveillance and the journal is not responsible for the maintenance of any links or email addresses provided therein.

S1 Supplementary methods and data description

S1.1 Methods and data

We analyse the qualitative outcome of the SARS-CoV-2 VirSNiP E484Q/K and L452R assay from Tib Mol Biol (Berlin, Bermany) performed on samples that tested positive for SARS-CoV-2 in partner laboratories, mainly using partly the PerkinElmer SARS-CoV-2 Real-time RT-PCR Assay (Perkin Elmer).

The VirSNiP test targets three mutations specifically (E484K, E484Q, and L452R). Based on the genomics of the different lineages and their prevalence in France, the test results are interpreted as follows:

- E484K-/E484Q-/L452R-: potential infection by the α variant
- E484K-/E484Q-/L452R+: potential infection by the δ variant
- E484K+/E484Q-/L452R-: potential infection by the β, γ, or η variant

All the other combinations were rare and grouped in the "other" category (see below for the details).

The data was extracted on June 28, 2021 and the most recent test results were from June 26, 2021. However, to correct for potential delays in the recording of the results from local partners, we did not include any tests performed after June 21, 2021. We also only included from individuals from 5 to 80 years old, and included at most one sample per individual.

The statistical methods are described in details in [1]. The data and R script used will be provided upon publication.

S1.2 Data characteristics

The characteristics of the data are shown in Table S1.

Among the ‘other’ test results, the majority corresponded to uninterpretable test results (751/1394, i.e. 54%) and a large fraction to tests without the L452R mutant and an ambiguity for the E484 mutation (524/1394, i.e. 38%), which could be indicative of an infected by a β, γ, or η variant with a low virus load. Note that this assumption is conservative to avoid overestimating the spread of the δ variant. In fact, some of the tests in the ‘other’ category (74/1394, i.e. 5%) find a clear signal for the L452R mutation with an ambiguity regarding the E484 mutation and could, therefore, be associated with this variant.

S1.3 Epidemiological model

The epidemiological model used to simulate the scenarios shown in Figure 2 is based on the discrete-timeframework adjusted to the French epidemic the methodology of which is detailed in [2]. The original model was extended to take into account the vaccine rollout, the extrapolation of which was based the

Table S1: Characteristics of the samples analysed via the VirSNiP assay, France, 31 May-21 June 2021 ($n = 9,030$).

**Characteristics**		**Value**
Age	*median [min,max]*	34 [6,84]
Sample origin	general population	8404 (93.1%)
	hospital	626 (6.9%)
Sampling date	*median [min,max]*	June 7 [May 31, June 21]
Region	Ile-de-France	4483 (49.9%)
	Normandie	1534 (17.1%)
	Hauts-de-France	1040 (11.6%)
	PACA	518 (5.8%)
	Centre-Val-de-Loire	415 (4.6%)
	other	993 (11.0%)
Test result	α-like	4478 (49.6%)
	$\beta/\gamma/\eta$-like	1165 (12.9%)
	δ-like	381 (4.2%)
	other	1235 (13.7%)
	uninterpretable	1771 (19.6%)

trend observed by the of June. We made the simplifying (and optimistic) assumption that the vaccine prevents 90% of critical infections and 80% of secondary infections directly from the first dose injection, while we assumed natural immunity to prevent 84% of reinfections [3], regardless of the virus genotype.

The modelled spread of the δ variant was based on a initial 25%-frequency on Jun 29th and a 70% transmission advantage compared to the α variant, assumed itself to have a 40% transmission advantage with respect to the historical strain [1].

References

[1] Roquebert B, Trombert-Paolantoni S, Haim-Boukobza S, Lecorche E, Verdurme L, Foulongne V, et al. The SARS-CoV-2 B.1.351 lineage (VOC β) is outgrowing the B.1.1.7 lineage (VOC α) in some French regions in April 2021. Eurosurveillance. 2021;26(23):2100447.

[2] Sofonea MT, Reyné B, Elie B, Djidjou-Demasse R, Selinger C, Michalakis Y, et al. Memory is key in capturing COVID-19 epidemiological dynamics. Epidemics. 2021;p. 100459.

[3] Hall VJ, Foulkes S, Charlett A, Atti A, Monk EJM, Simmons R, et al. SARS-CoV-2 infection rates of antibody-positive compared with antibody-negative health-care workers in England: a large, multi-centre, prospective cohort study (SIREN). The Lancet. 2021;397(10283):1459–1469.

S2 Supplementary Figure

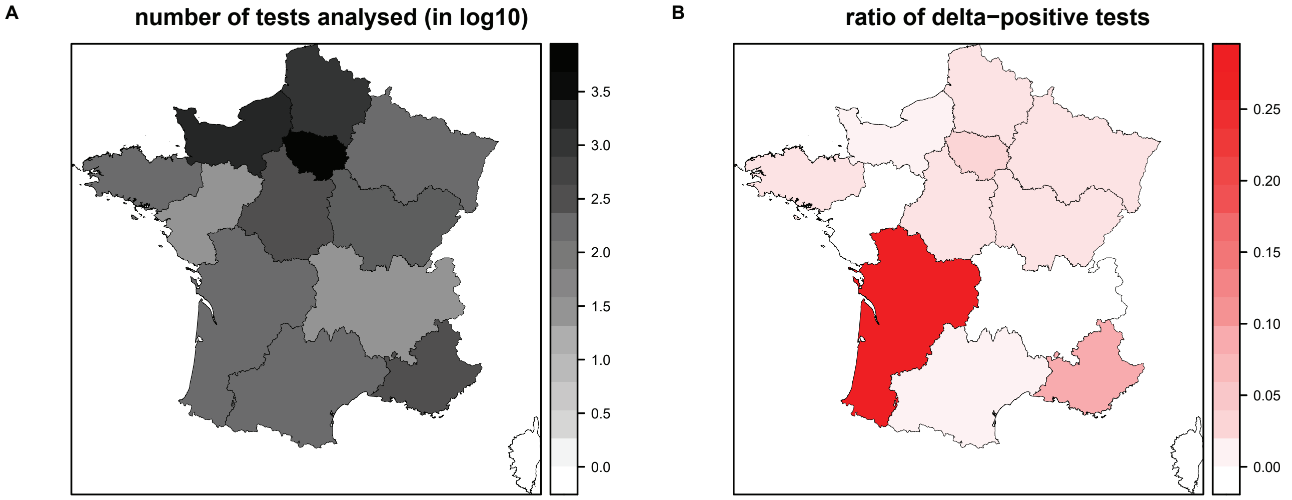

Figure S1: A) Number of tests analysed in French regions and B) Proportion of the tests consistent with an infection by the δ variant

S3 Supplementary Figure

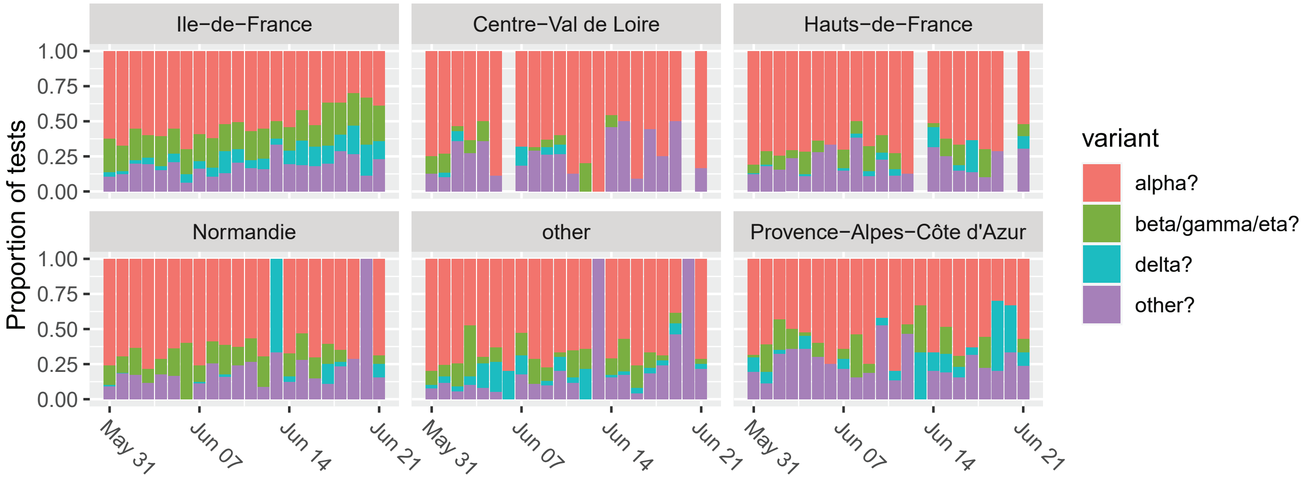

Figure S2: Proportion of tests associated with the main variants in the most densely sampled French regions.

S4 Supplementary Figure

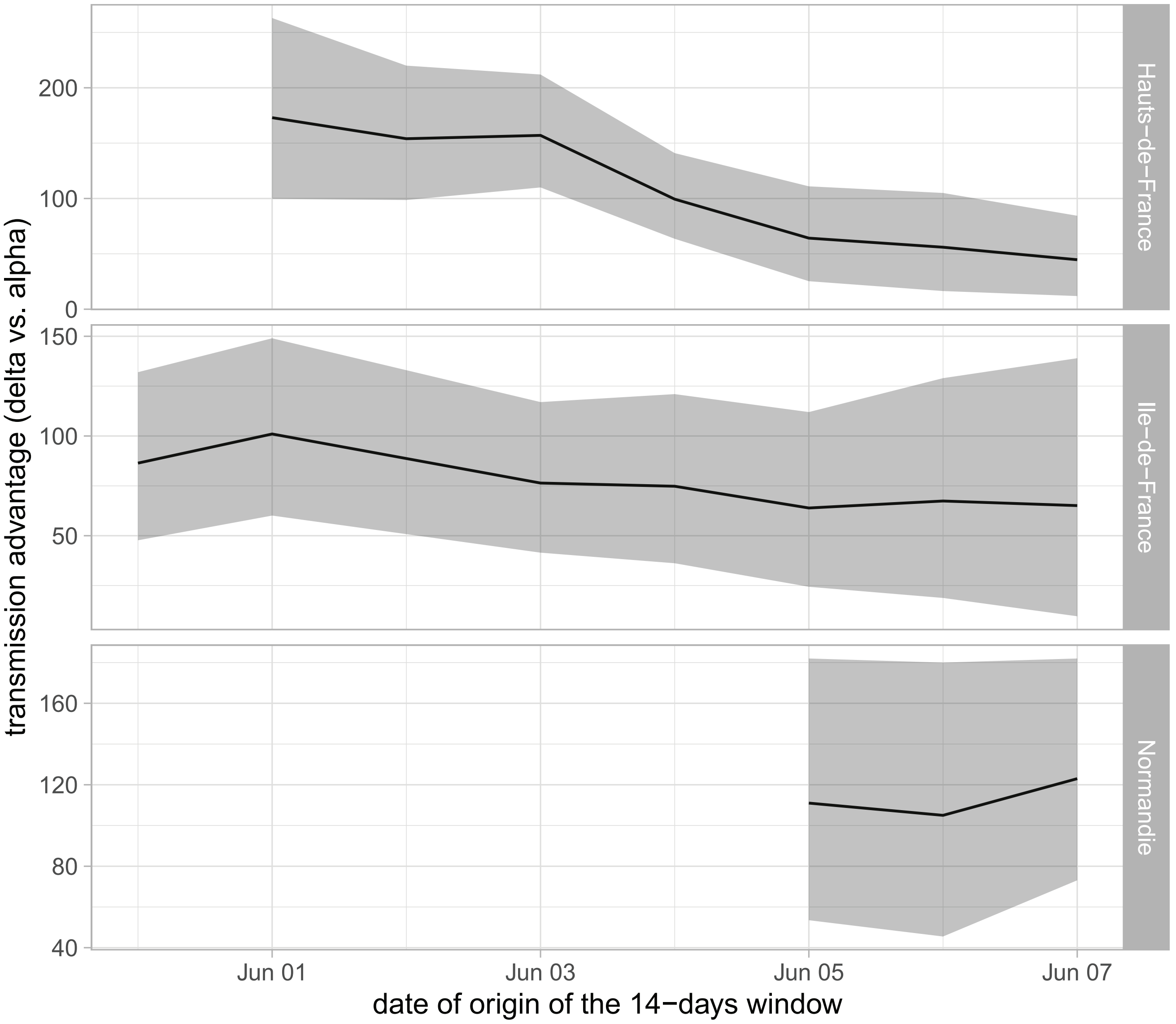

Figure S3: Sliding 14-days window estimating the transmission advantage of the δ variant of the α variant in 3 French regions.

S5 Supplementary Figure

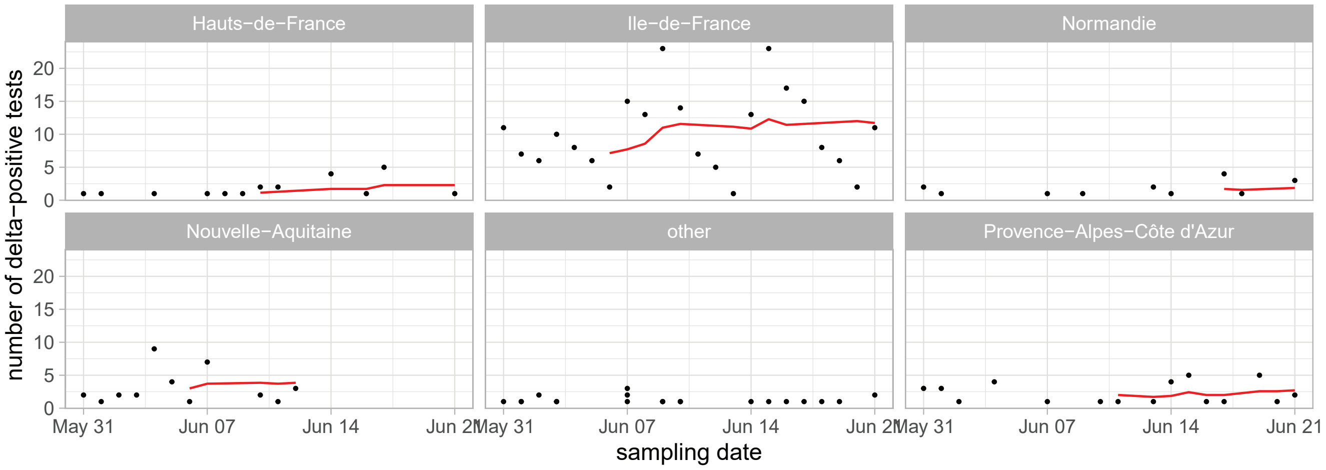

Figure S4: A) Number of tests consistent with an infection by the δ variant. The red line shows a right-aligned 7-day rolling mean.

S6 Supplementary Figure

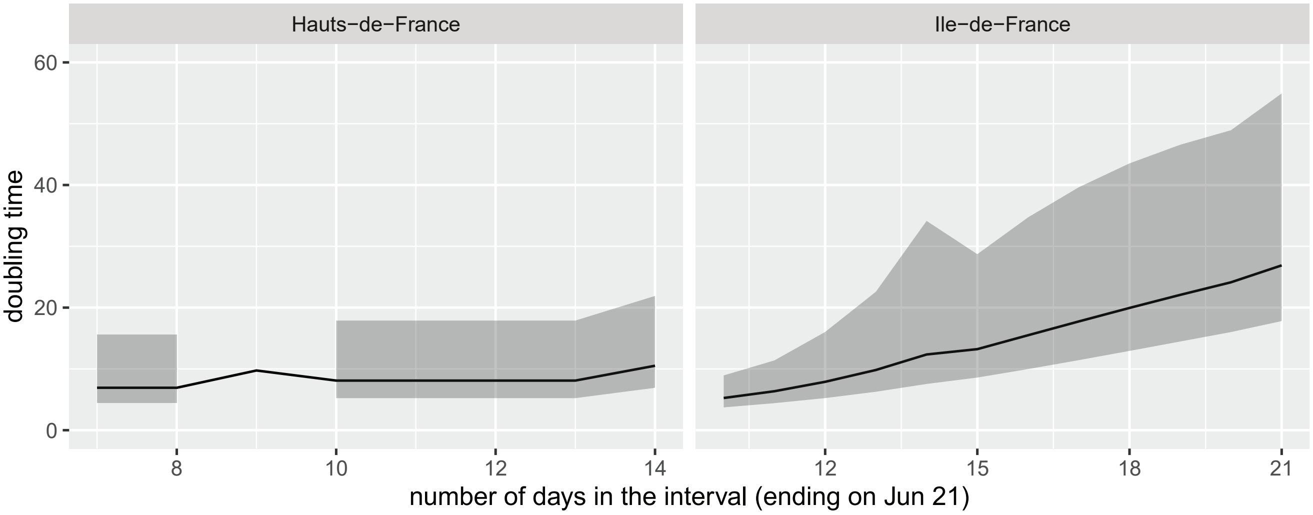

Figure S5: Epidemic doubling time as a function of the interval considered. The interval starts on May 31 in Ile-de-France and on June 7 in Hauts-de-France (due to a lack of data). The confidence interval are calculated using the regression model between the log of the number of cases and the time.
